# Correlates of severe disease in patients admitted with 2009 pandemic influenza A (H1N1) infection in Saurashtra region, India

**DOI:** 10.4103/0972-5229.74169

**Published:** 2010

**Authors:** Rajesh K. Chudasama, Pramod B. Verma, Chikitsa D. Amin, Bharat Gohel, Dinkar Savariya, Rakesh Ninama

**Affiliations:** **From:** Department of Community Medicine, Government Medical College, Rajkot, Gujarat, India

**Keywords:** Epidemiology, influenza A (H1N1), intensive care, pregnancy, reverse transcriptase-polymerase chain reaction, severe disease

## Abstract

**Background:**

India reported its first case of 2009 pandemic influenza A (H1N1) virus infection in May 2009 and in Saurashtra region in August 2009. We describe the epidemiology and factors associated with severe and non-severe cases of 2009 influenza A (H1N1) infection reported in Saurashtra region.

**Materials and Methods:**

From September 2009 to February 2010, we observed 274 patients who were infected with 2009 influenza A (H1N1) virus and admitted in different hospitals in Rajkot city. Real-time reverse transcriptase-polymerase chain reaction (RT-PCR) testing was used to confirm infection. Factors associated with severe disease were determined by comparing with non-severe cases.

**Results:**

Out of 274 patients, 87 had severe disease (requiring intensive care or died) and 187 had non-severe diseases (admitted in wards and survived). The median age of severe disease patients was 30 years; the median time was 5 days from the onset of illness to diagnosis, and 4 days median time was reported for hospital stay. More than half of the patients (56.3%) were females, and 58.6% patients were residing in urban area (OR = 1.65, CI = 0.97–2.8), among severe disease patients. Significant association (*P* < 0.01) was reported among severe disease patients for delayed referral from general practitioner/physician after initial treatment. All patients received antiviral drug, but only 19.5% received the same within 2 days of illness. Presence of coexisting condition [odds ratio (OR) = 0.53, confidence interval (CI) = 0.31–0.90], mainly pregnancy (OR = 0.22, CI = 0.06–0.76), was strongly associated with severe disease.

**Conclusion:**

Delayed referral from general practitioner/physician, duration of antiviral treatment, and presence of coexisting condition (especially pregnancy) were responsible for intensive care or mortality in patients of severe influenza A (H1N1) illness.

## Introduction

In April 2009, the novel influenza A (H1N1) virus was first detected in Mexico[1] and then in the United States (US).[2,3] This was originally referred to as “swine flu” because many of the genes in this new virus were found in pigs in North America.[4] Further on, it has been found that this new virus has gene segments from the swine, avian and human flu virus genes. The scientists called this a “quadruple reassortant” virus and hence this new (novel) virus was christened “Influenza A (H1N1) virus”.[5,6] The World Health Organization (WHO) raised the pandemic level from 5 to 6, the highest level after the documentation of human to human transmission of the virus in at least three countries in two of the six regions of the world defined by the WHO.[7]

The first case of confirmed infection with the virus in India was documented in May 2009[8] but only few cases were reported till August 2009. After that, a large numbers of positive cases were reported throughout India. From Gujarat state, the first H1N1 positive confirmed case was reported in June 2009.[9] Saurashtra region, in the western part of Gujarat state, reported its first case in August 2009.[10] All patients with confirmed infection were quarantined in isolation ward to prevent spread in the general population. Although many individuals presented with mild, self-limited illness, and no signs of pulmonary involvement, some people required intensive care and received maximal life support measures.[11,12] Predicting disease and mitigating hazard in at-risk populations is an important aim of public health epidemiology, and in preparation for future waves of pandemic H1N1 influenza, determining the correlates of the severity of disease may be very important.[13] Initial reports have suggested that in addition to many of the previously known risk factors, underlying co-morbidities may be the risk factors for severe disease.[2–14] The objective of present study was to identify factors associated with severity of disease in 274 lab confirmed cases of pandemic H1N1 influenza, hospitalized in various hospitals of Rajkot city of Saurashtra region from September 2009 to February 2010.

## Materials and Methods

### 

#### Data sources

The Ministry of Health and Family Welfare, Government of India, started preparations for the management of infected patients as soon as the first case was reported in May 2009. Gujarat state (including Saurashtra region) participated in active surveillance for pandemic H1N1 as of August 2009. All those government and private hospitals having advanced intensive care units (ICUs) were involved in admitting and managing influenza A (H1N1) positive patients in Rajkot. A total of 274 patients were found positive and admitted in different hospitals of Rajkot from 1 September 2009 to 20 February 2010.

#### Categorization of Influenza A (H1N1) case

The Ministry of Health and Family Welfare, Government of India, had issued guidelines for categorization of influenza A (H1N1) cases[15] during screening for home isolation, testing treatment, and hospitalization as (1) Category A; (2) Category B (i) and Category B (ii); and (3) Category C. Patients with Category A and B were treated on outpatient basis (with or without antiviral treatment), while patients with Category C were admitted to the hospital and given antiviral treatment also. Present study included all patients of Category C.

#### Clinical case/suspected case definition

A suspected case was defined as one having influenza like illness (temperature ≥ 37.5°C with at least one of the following symptoms: sore throat, cough, rhinorrhea, or nasal congestion) and either a history of travel to a country where infection had been reported or epidemiologic link to a person with confirmed or suspected infection in the previous 7 days. A confirmed case was defined by a positive result of a real-time reverse transcriptase-polymerase chain reaction (RT-PCR) assay performed at a laboratory operated under the auspices of the state government.[8]

#### Criteria for ICU admission

All patients were categorized as (1) cases (severe influenza A (H1N1) patients): those patients who needed admission to ICUs or died. Patients with one or more of following features were admitted in ICU (a) SPO_2_ < 60 mm of Hg, (b) not maintaining SPO_2_ with oxygen mask, (c) tachypnea and dyspnea, (d) respiratory rate > 40/min, (e) with altered sensorium, (f) patchy consolidation on X-ray chest and (2) controls as non-severe influenza A (H1N1) patients: those admitted in wards, who survived and did not need intensive care. Patients not having any of the above criteria were admitted in wards for clinical management.

#### Variables

Several types of data were collected from the patients: date and time of admission to hospital/ICUs, age, sex, residential status, coexisting conditions, date and time of first symptoms. Also, other variables were collected from medical record and statistics department of hospitals, including presence and type of influenza syndrome, duration of treatment in hospitals and ICU, duration between onset of illness and diagnosis, whether kept on ventilatory support, outcome of hospital/ICU admission, time from onset of illness to death, and time from initiation of antiviral drug to death.

#### Data management

The admission history and medical records of all the patients were assessed from swine flu ward for initial clinico-epidemiological details, and from medical record and statistics department after patient discharge/death from various hospitals of Rajkot city. Line list number was given to every patient to avoid duplication at any time during the study period. Approval by institutional review board was not required because this infectious disease was covered under the epidemic act and the state health department[16] implemented the Epidemic Disease Control Act, 1897 on 18 August 2009 and issued a notification that it was in the interest of the public health to collect data on an emerging pathogen.

#### Laboratory confirmation of infection

The influenza A (H1N1) virus was detected using real-time RT-PCR assay in accordance with the protocol from the US centers for Disease Control and Prevention, as recommended by the WHO.[17] Two swabs from the naso-pharynx and one from the pharynx were collected from suspected patients and their contacts for detection of influenza A (H1N1) virus by real-time RT-PCR assay.

#### Statistical analysis

All the data were entered in MS Excel and analyzed by using Epi Info software (version 3.5.1) from CDC.[18] Bivariate analysis was done using χ^2^ test or Fisher’s exact test for analysis. Variables that showed *P* < 0.20 in bivariate analysis were selected for logistic regression to examine the relation between variables of interest and severity of disease. A comparison was made between patients who needed intensive care or died and patients who did not need intensive care and survived. Results from logistic regression analyses were expressed as odds ratio (OR), and 95% confidence intervals (CIs). The *P* values and CIs reported here reflect a two-tailed α level of 0.05.

## Results

### 

#### Demographic and clinical characteristics of patients

A total of 274 cases infected with H1N1 influenza A [Table 1] were diagnosed and hospitalized in different hospitals at Rajkot in 2009. Out of the 274 cases of influenza A (H1N1), 87 (31.8%) reported with severe disease and 187 patients (68.2%) with non-severe disease. Among the 87 patients with severe disease, mortality was reported in majority (81.6%) of the patients, while only 18.4% patients needed intensive care and survived.

**Table 1:** Baseline characteristics of 2009 influenza A (H1N1) infected patients in Saurashtra region from September 2009 to February 2010

Characteristics	Non-severe influenza A (H1N1) (n = 187)	Severe influenza A (H1N1) (n = 87)
Age in years		
Median	28 years	30 years
Range	(6 months–68 years)	(4 months–68 years)
Age group of patients (years) – no. (%)		
<15	41 (21.9)	18 (20.7)
15–24	35 (l8.7)	9 (10.3)
25–44	68 (36.4)	38 (43.5)
45–64	39 (20.9)	20 (23.0)
≥65	4 (2.1)	2 (2.3)
Sex – no. (%)		
Female	84 (44.9)	49 (56.3)
Male	103 (55.1)	38 (43.7)
Residential status – no. (%)		
Urban	131 (70.1)	51 (58.6)
Rural	56 (29.9)	36 (41.4)
First treated at general practitioner/physician	67 (35.8)	49 (56.3)
Hospital stays in days – no. (%)		
Median (in days)		
£2	7	4
3–5	11 (5.9)	30 (34.5)
6–10	50 (26.7)	22 (25.3)
≥11	100 (53.5)	15 (17.2)
	26 (13.9)	20 (23.0)
Time interval from onset of illness to hospital admission and diagnosis – no. (%)		
Median (in days)	5	5
<1	10 (5.3)	8 (9.2)
1–4	83 (44.4)	32 (36.8)
5–10	88 (47.1)	45 (51.7)
>10	6 (3.2)	2 (2.3)
Antiviral treatment received – no. (%)	187 (100)	87 (100)
≤2 Days after onset of symptoms	27 (14.4)	17 (19.5)
Patients kept on ventilators – no. (%)	0	70 (80.5)
Median duration on ventilators	0	3 days
Hospital outcome		
Intensive care and survived	0	16 (18.4)
Intensive care and died		
	0	71 (81.6)

**Figure 1:**
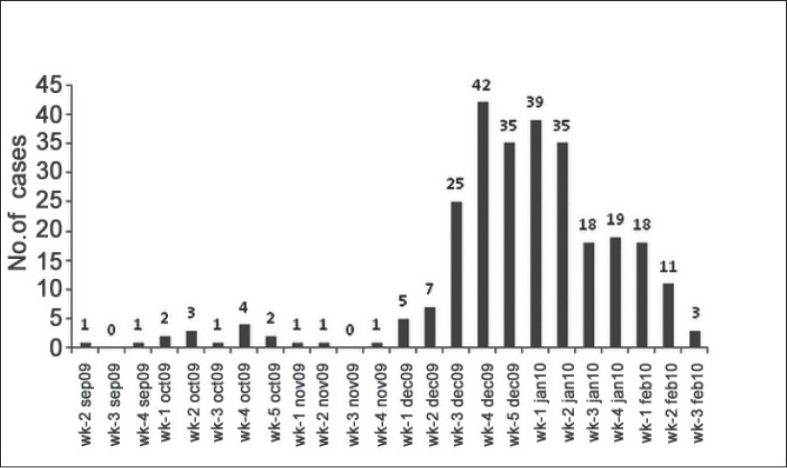
Week-wise distribution of influenza A (H1N1) infected hospitalized patients

Week-wise distribution [Figure 1] showed that number of cases increased gradually from the first week of December 2009. From the third week of December, a sudden increase was reported, with the highest positive cases (*n* = 42) in the fourth week of December 2009. It remained high during January 2010, which was followed by a gradual fall in February 2010.

The median age was 30 years (range 4 months to 68 years) in severe disease patients and 28 years (range 6 months to 68 years) in non-severe patients. More females (56.3%) (OR = 0.63, CI = 0.37–1.05) needed intensive care than males. Significant number of patients from urban areas reported severe disease (*P* < 0.05). The median duration of diagnosis of infection was 5 days after the onset of illness (range 1–20 days) for patients of both the groups. They reported mainly cough, fever, sore throat and shortness/difficulty in breathing [Table 2]. A total of 91 (33.2%) cases had an underlying medical condition [Table 2], reported significantly among severe disease patients (42.5%) (*P* < 0.05). Diabetes mellitus (DM) (11.8%) and/or hypertension (10.2%) was the mainly reported underlying condition among non-severe disease patients, but pregnancy (11.5%) (*P* < 0.05) was reported mainly among patients with severe disease [Table 2]. Among the female patients who reported, 15 (5.5%) were pregnant with a range of 5–9 months of amenorrhea and significant risk of severe disease was reported with pregnancy (OR = 0.22, CI = 0.06-0.76).

**Table 2:** Clinical features and coexisting conditions among influenza A (H1N1) infected patients

Characteristics	Severe influenza A (H1N1) (*n* = 87)	Non-severe influenza A (H1N1) (*n* = 187)
Clinical features – no. (%)		
Cough	85 (97.7)	180 (96.3)
Fever (>37.5°C)	81 (93.1)	171 (91.4)
Sore throat	44 (50.6)	105 (56.1)
Shortness/difficulty in breathing	50 (57.5)	96 (51.3)
Nasal catarrh	19 (21.8)	49 (26.2)
Headache	25 (28.7)	35 (I8.7)
Vomiting	21 (24.1)	26 (13.9)
Coexisting conditions – no. (%)		
Any one condition*	37 (42.5)	53 (28.3)
Diabetes mellitus	5 (5.7)	22 (11.8)
Hypertension	5 (5.7)	19 (I0.2)
Chronic pulmonary diseases	2 (2.3)	13 (7.0)
Pregnancy*	10 (11.5)	5 (2.7)
Chronic heart diseases	4 (4.6)	9 (4.8)
Seizure disorder	2 (2.3)	5 (2.7)
Chronic renal failure	0	2 (1.1)

* *P* < 0.05

#### Laboratory and radiographic findings

Leukopenia (27.4%), anemia (41.9%), lymphopenia, and thrombocytopenia (29%) were reported in patients with severe disease [Table 3]. Pneumonia was reported more among patients with severe disease (96%) (OR = 0.69, CI = 0.34–1.41) [Table 4].

**Table 3:** Laboratory and radiological findings of influenza A (H1N1) infected patients*

Characteristics	Severe influenza A (HINI) No./total no. (%)	Non-severe influenza A (HINI) No./total no. (%)
Leukocyte count		
Mean count	7869 ± 6991	7099 ± 3942
Leukopenia (<4000/mm^3^)	20/73 (27.4)	39/165 (23.6)
Leukocytosis (>10,000/mm^3^)	19/73 (26.0)	32/165 (19.4)
Hemoglobin g/dl	11.27 + 2.66	11.65 ± 2.49
Anemia		
Mild (10.0–11.0 g/dl)	10/74 (13.5)	15/166 (9.0)
Moderate (8–10 g/dl)	13/74 (17.6)	22/I66 (13.3)
Severe (<8 g/dl)	8/74 (10.8)	15/166 (9.0)
Lymphocyte count		
<1500/mm^3^ in adults	42/53 (79.2)	76/125 (60.8)
<3000/mm^3^ in children	3/15 (20.0)	7/39 (17.9)
Platelet count		
Mean count	231,834 ± 115,130	214,333 ± 138,592
Thrombocytopenia (<150,000/mm^3^)	20/69 (29.0)	29/145 (20.0)
Thrombocytosis (>350,000/mm^3^)	8/69 (11.6)	19/145 (13.1)
Elevated alanine aminotransferase (>40 U/l)		
Any deviation	32/36 (88.9)	67/79 (84.8)
≥2× the upper limit of normal range	30/36 (83.3)	60/79 (75.9)
Elevated aspartate aminotransferase (>40 U/l)		
Any deviation	12/32 (37.5)	24/78 (30.8)
≥2× the upper limit of normal range	2/32 (6.3)	I0/78 (I2.8)
Elevated total bilirubin (>1.2 mg/dl)	10/42 (23.8)	23/94 (24.5)
Erythrocyte sedimentation rate		
>I5 mm/hr in male patients	10/25 (40.0)	33/67 (49.3)
>20 mm/hr in female patients	7/25 (28.0)	19/67 (28.4)
Chest X-ray findings		
Done	75/87 (86.2)	152/187 (81.3)
Pneumonia foundt	72/75 (96.0)	139/152 (91.4)
Antibiotic treatment received	79/87 (90.8)	163/187 (87.2)
Corticosteroid treatment received	54/87 (62.1)	72/187 (38.5)

* ± values are mean ± SD †*P* < 0.05

**Table 4:** Correlates of disease severity among severe (*n* = 87) and non-severe (*n* = 187) influenza A (H1N1) patients

Characteristics	Severe influenza A (HINI) No. (%)	Non-severe influenza A (HINI) No. (%)	*P* value	Odds ratio (OR)	95% Confidence interval
Age group: ≤ 15 years vs. > 15 years	19 (21.8)	43 (23.0)	0.83	—	—
Sex: female vs. male	49 (56.3)	84 (44.9)	0.07	0.63	0.37–1.05
Residential status: Urban vs. rural	51 (58.6)	131 (70.1)	0.06	1.65	0.97–2.80
First treated by a general practitioner/physician	49 (56.3)	67 (35.8)	0.00	0.43	0.25–0.72
Hospital stay in days: ≤ 5 days vs. > 5 days	35 (40.2)	126 (67.4)	0.00	0.32	0.19–0.55
Time from onset of illness to diagnosis: ≤ 5 days vs. > 5 days	58 (66.7)	134 (71.7)	0.40	—	—
Interval from symptom onset to antiviral treatment: < 2 days vs. ≥ 2 days	17 (19.5)	27 (14.4)	0.28	—	—
Time from antiviral drug started to outcome: <5 days vs. ≥ 5 days	79 (90.8)	161 (86.1)	0.00	2.88	1.45–5.70
Pneumonia	75 (86.2)	152 (81.3)	0.16	0.69	0.34–1.41
Presence of any coexisting condition	37 (42.5)	53 (28.3)	0.021	0.53	0.31–0.90
Pregnant females (females aged 18—45 years)	10 (11.5)	5 (2.7)	0.01	0.22	0.06–0.76

#### Treatment outcome

More than half of the patients with severe disease (56.3%) and 35.8% of the non-severe disease patients were first treated by a general practitioner/physician and then referred to the higher center (OR = 0.43, CI = 0.25-0.72). The median time for hospital stay was found to be 7 days for influenza A patients (H1N1) in non-severe cases, while it was 4 days for severe cases. Five days median time from onset of illness to diagnosis and hospitalization was reported in both the categories. Among the 87 severe disease patients, 81.6% of patients who needed intensive care, reported mortality and 18.4% survived.

All the patients had received antiviral drug oseltamivir [Table 1]. Out of the 87 severe disease patients, 19.5% received the antiviral drug within 2 days of onset of illness. Median time of 3 days for ventilatory support, more than 5 days hospitalization (*P* < 0.05) and antiviral drug administration (*P* < 0.05) were obtained among severe disease patients.

Majority of the patients who needed intensive care and died were females. Also, many of the patients were residing in an urban area and had a coexisting condition (OR = 1.65, CI = 0.97–2.80), especially pregnancy (OR = 0.22, CI = 0.06–0.76). The mean hospital stay for patients who needed intensive care was 5 days or more which was higher than that for the patients who did not need intensive care. They were also more likely to receive oseltamivir for 5 days or more and corticosteroids [Table 4].

## Discussion

This study focused on severe influenza A (H1N1) virus infection in residents of Saurashtra region. They were associated with longer interval from the onset of symptoms to treatment with antiviral therapy and with the presence of coexisting conditions than among non-severe patients. This study identified all patients with confirmed 2009 influenza A (H1N1), belonging to category C,[15] hospitalized in various hospitals in Rajkot from September 2009 and February 2010. A total of 274 patients reported to the hospitals, were confirmed and hospitalized during the study period and were categorized as patients having severe disease (*n* = 87) and non-severe disease (*n* = 187).

The median age of patients with severe disease was found to be 30 years, higher than that reported in China (23.4 years),[19] but lower than that reported in Canada (33.4 years).[13] Two-thirds of the patients with severe disease were above the age group of 25 years, and 56.3% were females. It indicates that adults and females[13] (OR = 0.63, CI = 0.37–1.05) appear to be at a higher risk of death due to pandemic influenza A (H1N1) virus infection compared to children or teenagers. Severe influenza cases were reported more from the urban area (OR = 1.65, CI = 0.97–2.80) than rural area,[13] which may be due to the dense population in urban area favoring spread of virus infection. A median time of 5 days was reported from the onset of illness to diagnosis of influenza A (H1N1) among all patients. More than half (56.3%) of the patients with severe disease were treated first by a general practitioner/physician (OR = 0.43, CI = 0.25–0.72) and then referred to a higher center. The time duration between onset of illness and hospital admission and diagnosis was more than that reported from other countries.[14,20] The possible justification is that patients seek treatment at a local level from general practitioners and physicians, but with no or little improvement after initial treatment, they are referred to a higher center for further investigation and management. The present study reported a median time of 4 days for hospital stay among the severe disease patients (OR = 0.32, CI = 0.19–0.55) with 60% patients having less than 5 days hospital stay, compared to 7 days median time and 33% having less than 5 days hospital stay in non-severe disease patients. It also indirectly reflects that patients with more severe disease with delayed referral approach a higher center at a critical stage.

Majority of the 2009 H1N1 viruses that have been tested at the CDC to date have been susceptible to two neuraminidase inhibitors, oseltamivir and zanamivir, and resistant to two adamantanes, amantadine and rimantadine.[6,21] Current interim CDC guidelines for pandemic and seasonal influenza recommended the use of either oseltamivir or zanamivir for hospitalized patients with suspected or confirmed influenza and for outpatients who are at high risk for complications.[22] The Ministry of Health and Family Welfare, Government of India, has recommended and supplied oseltamivir to the state governments for distribution in tertiary care centers and district hospitals in adequate quantity and was available in the reported region also. Although the evidence of benefit from antiviral therapy was the strongest when treatment was initiated within 48 hours after the onset of illness, a study with oseltamivir in hospitalized patients reported reduction in mortality even after 48 hours of onset of illness.[23] In the present study area, all the influenza A (H1N1) infected fatal cases received oseltamivir after hospital admission, but only 19.5% severe disease patients received it within 2 days of onset of illness, while in the United States, 45% infected patients received oseltamivir within 2 days of onset of illness.[20] Initial primary treatment at general practitioners or local physician level and delayed referral to higher center and investigation may be the possible explanation for delayed start of oseltamivir in suspected or confirmed influenza A (H1N1) patients. When started early, the antiviral drug has a beneficial effect. A study reported that patients admitted to ICU or died were less likely to have received such therapy within 48 hours after onset of symptoms.[20] Present study suggests 90% mortality in severe disease patients even after complete course of oseltamivir therapy (OR = 2.88, CI = 1.45–5.70), possibly because of delayed referral and initiation of antiviral drug.

Week-wise distribution [Figure 1] of influenza A (H1N1) infected patients in Saurashtra region shows that number of cases increased gradually from the first week of December 2009. By the third week of December 2009, a sudden increase was reported, with highest positive cases (*n* = 42) in the fourth week which remained at a high level during January 2010, followed by a gradual fall in the number of positive cases in February 2010. In India, the monsoon ends by September and October, which is followed by start of winter from November. The atmospheric temperature remains lowest in December, correlating with an increase in the reported number of infected patients with influenza A (H1N1). It continues in January and the winter comes to an end by February; the number of reported positive cases also shows a fall. It signifies the relationship of influenza virus with cold season as maximum number of cases presented during these months of winter season, as reported by other studies.[12,14,20]

Present study shows that majority of the patients in both categories had cough, fever, shortness of breath, and sore throat, similar to patients from United States,[20] Canada,[13] Australia and New Zealand.[14] Current study shows that 42.5% severe influenza A (H1N1) patients have any one coexisting condition (OR = 0.53, CI = 0.31–0.90), which was 36% in England,[24] and 53% in France.[25] Pregnancy was a well-documented risk factor for severe infection and death in seasonal influenza and in previous pandemics.[26–28] In this study, pregnancy as a risk factor (OR = 0.22, CI = 0.06–0.76) was reported in 11.5% severe influenza A (H1N1) cases than among non-severe influenza A (H1N1) cases.[24,25,29] Out of 10 severe disease pregnant cases, 2 were in the second trimester and 8 were in the third trimester.

Pneumonia was reported more among patients with severe disease (96%) (OR = 0.69, CI = 0.34–1.41) than among non-severe patients. All hospitalized patients with evidence of pneumonia received antiviral drugs and antibiotics, which was higher than in the patients from the United States (73%).[20] In the absence of accurate diagnostic methods, patients who were hospitalized with suspected influenza and lung infiltrates on chest radiography should be considered for treatment with both antibiotics and antiviral drugs.[3]

Our study has a number of strengths. It represents one of the largest series of hospitalized cases with severe 2009 influenza A (H1N1) infection, covering two seasons of monsoon and winter. It includes both adults and children from geographically similar areas, which improves the generalizability of our results to other regions. These observations of epidemiological risk factors, typical clinical features, response to therapy, and prognosis should aid in the recognition, diagnosis and clinical management of influenza A (H1N1).

### 

#### Limitations

Our study also has some limitations. The data were taken from only hospitalized patients, so patients who got infected in the community and did not go to the hospital were not included in our study. Also, patients belonging to category B (i) or B (ii) who were treated on outpatient basis and not being tested were not included in present study. All diagnostic testing was clinically driven, and other investigations were not obtained in a standardized fashion. Despite the use of a standardized data collection form, not all information was collected for all patients.

We were also unable to assess the factors relating to education level or household size. Considering association between coexisting condition and severity of disease, it is possible that the presence of a coexisting condition that makes ICU admission more likely might also have made ascertainment of virologic infection more likely, thus producing an inflated estimate of any potential association. With regard to present study, the relative impact of the direction of this type of selection bias, known as Berksonian bias, is uncertain. The overall findings may be different during future waves, owing to the timely deployment of an effective vaccine, to viral mutation, and resistance to antiviral drugs.

## Conclusion

The severity of illness among influenza A (H1N1) infected patients was associated more with delayed referral from general practitioner/physician, duration of antiviral treatment, presence of coexisting condition (especially pregnancy), than non-severe influenza A (H1N1) infected patients. These findings may be different during future waves, owing to the timely deployment of an effective vaccine, to viral mutation, and resistance to antiviral drugs.
